# Roles of CXCL5 on migration and invasion of liver cancer cells

**DOI:** 10.1186/1479-5876-12-193

**Published:** 2014-07-10

**Authors:** Xiaojing Xu, Peixin Huang, Biwei Yang, Xiangdong Wang, Jinglin Xia

**Affiliations:** 1 Liver Cancer Institute, Zhongshan Hospital, Fudan University; Key Laboratory of Carcinogenesis and Cancer Invasion, Ministry of Education, Shanghai 200032, China; 2 Department of Pulmonary Medicine, The First Hospital of Wenzhou Medical University, Wenzhou, China

**Keywords:** CXCL5, CXCR2, Migration, Invasion, Liver cancer

## Abstract

Inflammatory factors play a vital role in the progression of liver cancer, although exact factors and related mechanisms still remain unclear. The present study aimed at screening inflammatory factors related to liver cancer metastasis and investigating the potential mechanism by which cancer cells are recruited. We screened and validated inflammatory factors by microarray and RT-PCR. Small interfering RNA (siRNA) and recombinant protein were used to assess CXCL5 effects on the movement of liver cancer cells (LCCs). Our screening microarray demonstrated over-expression of CXCL5 in LCCs with high metastatic potentials. CXCL5 increased LCCs migration and invasion, probably through autocrine and paracrine mechanisms. CXCL5-CXCR2 and ERK1/2 pathways could play critical roles in the regulation of LCCs migration. Our data indicates that LCCs per se may act as the producer and receptor of CXCL5 responsible for liver cancer migration and invasion.

## Introduction

Primary liver cancer is the fifth most common malignancy and the third commonest cause of cancer mortality [1,2]. Metastasis is one of the main characteristics of primary liver cancer, contributing to a poor 5-year survival rate (<9%) [3]. Inflammatory factors were proposed to play an important role in the metastasis of liver cancer cells (LCCs) [4,5]. Cancer cells could produce a variety of inflammatory factors to chemo-attract leukocytes from the circulation to tumor tissues [6,7]. Recruited leukocytes or activated cancer cells could further release inflammatory mediators to regulate tumor metastasis, although the exact factors and related mechanisms remain unclear.

Chemotaxis of cancer cells and stromal cells in the microenvironment is an essential component of tumor dissemination during metastasis. Epithelial neutrophil-activating peptide-78 (ENA-78/CXCL5) is a CXC chemokine with presence of ELR motif at the NH2 terminus. ELR-CXC chemokines have been proposed as important mediators of tumorigenesis in a number of cancers [8] and CXCL5 was also found over-expressed in many types of cancers, including liver cancer, mediating neutrophil infiltration and indicating poor prognosis [9-13]. However, further studies about the role of CXCL5 on the recruitment of liver cancer cells are still necessary.

The present study aimed at screening inflammatory factors related with metastasis of liver cancer and investigating the potential mechanism by which they were involved in cancer cell recruitment. We initially selected epithelial CXCL5 as the target from gene mapping result to investigate its chemo-attractive roles in the tumor cell recruitment. Furthermore, the CXCL5-CXCR2-ERK signaling pathway in LCCs was also monitored.

## Methods

### Reagents

Human CXCL5/ENA-78 quantikine enzyme-linked immunosorbent assay (ELISA) kit (DX000), recombinant human CXCL5/ENA-78, and anti-CXCR2/CXCL8RB and anti-CXCL5 were purchased from R&D (Minneapolis, MN, USA). SYBR Premix Ex Taq was from TaKaRa (Shiga, Japan). Matrigel™ Basement Membrane Matrix was from BD Bioscience (Franklin Lakes, NJ, and USA). Lipofectamine™ 2000 Transfection Reagent was from Invitrogen (Grand Island, NY, USA). Anti-p44/42 MAPK(Erk1/2), anti–phospho-p44/42 MAPK(Erk1/2) (Thr202/Tyr204), anti-p38 MAPK, anti–phospho-p38 MAPK (Thr180/Tyr182), anti-SAPK/JNK, anti–phospho-SAPK/JNK (Thr183/Tyr185), and ERK1/2 inhibitor U0126 were from Cell Signaling Technology (Boston, MA, USA). CXCR2 inhibitor SB225002 was obtained from Calbiochem (Darmstadt, Germany).

### Cell lines

Our institute established human LCCs with high metastatic capacity (HCCLM3, MHCC97H and MHCC97L) [14,15] with seeding density of 2 × 10^4^/cm^2^. LCCs with low metastatic capacity (SMCC7721, HepG2) were from ATCC cell bank, and the seeding density was 4 × 10^3^/cm^2^. Cells were grown in Dulbecco's modified Eagle's medium or RPMI-1640 supplemented with 10% fetal bovine serum (FBS, Hyclone) at 37°C in a 5% CO_2_, 95% air environment in humidified incubators.

When the cell density grew to 80-90%, mRNA and cell supernatant were collected for RT-PCR and ELISA respectively.

### Mapping of inflammatory genes

Expressions of inflammatory genes were evaluated by the human RT^2^*Profiler* PCR Inflammatory Cytokines and Receptors Array (catalog number: PAHS-011, SABiosciences). Total RNA was isolated using TRIZOL™LS reagent (Invitrogen, Carlsbad, CA). Two micrograms of RNA were used for cDNA synthesis with the RT^2^ First Strand Kit (SABiosciences). The RT^2^*Profiler* array was probed according to the manufacturer’s protocol using the Profiler PCR Array System and SYBR Green/Fluorescein qPCR Master Mix (SABiosciences) in an ABI 7900 sequence analyzer (Applied Biosystems). Gene expressions were compared with the dedicated Web-based software package (http://pcrdataanalysis.sabiosciences.com/pcr/arrayanalysis.php), which automatically performs all 2^-ΔCt^ based fold-change calculations from the specific uploaded raw threshold cycle data [ΔCt = Ct (inflammatory genes) - Ct (beta-actin)]. Differential expression values were identified using analysis of variance and/or Student *t* test with a significance value of *P* < 0.05 and a fold-change cut-off of 2-fold.

### Measurement of mRNA expression

RNA isolation was performed using the TRIZOL™LS reagent (Invitrogen, Carlsbad, CA). The cDNA was prepared using an oligo (dT) primer (Additional file 1: Table S1, not shown) and reverse transcriptase (Takara, Shiga, Japan) following standard protocols. Quantitative real time polymerase chain reaction (qRT-PCR) was performed using SYBR Green on the ABI 7500 real-time PCR System (Applied Biosystems, Foster City, CA). Each PCR reaction mixture contained 10 μM of each primer, 10 μl of 2 × SYBR Green Premix Ex Taq, 1.6 μl cDNA and RNase-free water, with a total volume of 20 μl. The PCR reaction was carried out with a denaturation step at 95°C for 10 mins, then 45 cycles at 95°C for 10 sec and finally at 60°C for 20 sec. All PCRs were performed in triplicate and normalized to internal control beta-actin mRNA. Relative expression was presented using the 2^-ΔCt^ method [ΔCt = Ct (chemokine) - Ct (beta-actin)].

### Measurements of CXCL5 production

Levels of CXCL5 protein in the cell supernatant were determined using ELISA in accordance with the protocol provided by the manufacturer. Briefly, samples and standards were added in a 96 well polystyrene microplate coated with CXCL5 primary antibody and incubated for 2 hrs. The plates were washed, added with CXCL5 conjugate antibody, and incubated for 2 hrs. The substrate solution was added for color development after washing twice, and the reaction was terminated with stop solution. Absorbance was measured at 450 nm. The final cell number was counted and the amount of protein secreted by 10^3^ cells was used to represent the expression levels of various cells.

### Immunocytochemistry

Cells were fixed with 4% paraformaldehyde for 30 minutes and permeabilized with wash buffer with 0.5% Triton X-100 and 0.01% sodium azide. Cells were blocked with 1% bovine serum albumin for 30 minutes, incubated in CXCR2 primary antibody or PBS as controls overnight, and then incubated with secondary antibody after washing thrice. Diaminobenzidine was added and incubated for 5 minutes. Slips were stained with hematoxylin and placed with mounting medium and scanned with an Olympus confocal microscope (at 200× magnification).

### Small interfering RNA transfection

Small interfering RNA (siRNA) transfections were performed, according to the manufacturer’s protocol, in 6-well plates using Lipofectamine™ 2000 with three different sequences of siRNA (Additional file 1: Table S1) duplexes targeting CXCL5 and a double-stranded RNA negative control (GenePharma, Shanghai, China). Three μl of Lipofectamine™ 2000 and 60 pmol of each siRNA were transfected in triplicate, except for ratio-dependent effect studies where several ratios of Lipofectamine™ 2000/siRNA were tested to optimize the efficacy of transfection. Cells were prepared for quantitative RT-PCR and ELISA analyses either 48 hrs or 72 hrs after transfection.

### Western blot

Protein samples (50 μg) were mixed with one-fourth volume of SDS sample buffer, boiled for 5 mins, and then separated through 10% SDS-PAGE gels. After electrophoresis, proteins were transferred to nylon membranes by electrophoretic transfer. Membranes were blocked in 5% bovine serum albumin for 1 hr, rinsed and incubated with primary antibodies in TBS diluted at 1:1000 at 4°C overnight. Primary antibody was then removed by washing in TBS-tween thrice, and labeled by incubating with 0.1 mg/ml peroxidase-labeled secondary antibodies against the mouse or rabbit for 1 hr. Bands were visualized by electrochemiluminescence and exposed to X-ray film following washing thrice in TBS-tween.

### Migration and invasion assay

Transwell chamber inserts (Corning Inc, Corning, NY) with filter membrane pore size of 8 μm were coated with 80 μL Matrigel (0.8 mg/mL, BD Bioscience, Mountain View, CA). HepG2 and MHCC97H cells were incubated on the upper chamber at the concentration of 5 × 10^5^ /mL in serum-free DMEM. DMEM containing 10% FBS was added to the lower compartment with recombinant CXCL5 at final concentrations of 0.1, 1.0, or 10 nM or with cell culture supernatant after transfection. Cells migrated through the permeable membrane during 48 hrs were fixed in paraformaldehyde and stained with Giemsa. Cells in five microscopic fields (at 200× magnification) were counted and photographed. Each assay was done at least in triplicates. Migration assays were applied similarly without coating the upper chamber with Matrigel and migrated cells were counted at 24 hrs. To investigate the role of CXCR2 or ERK1/2 pathway, cells were pretreated with CXCR2 inhibitor SB225002 at 5 or 10 μg/ml or ERK1/2 inhibitor U0126 at 5 or 10 μM for 2 hrs.

### Wound-healing assay

GFP-HepG2 cells were seeded in 6-well plates and incubated for 12 hrs. Two intersecting lines were scratched by a sterilized pin to wipe off the adherent cells in these lines to create a wound. SB225002 or U0126 were then added to the medium and a control was set up with an equal concentration of DMSO. The migration of HepG2 was assessed using an inverted light microscope at the original magnification X100 and migrated distances of HepG2 were measured at 0, 24 or 48 hrs.

### Statistical analysis

Data was expressed as mean ± standard errors. Statistical comparisons of the results were made using analysis of variance (ANOVA). Significant differences (P < 0.05) between groups were analyzed by LSD test.

## Results

### Expression of inflammatory factors/receptors in LCCs

**Figure 1:**
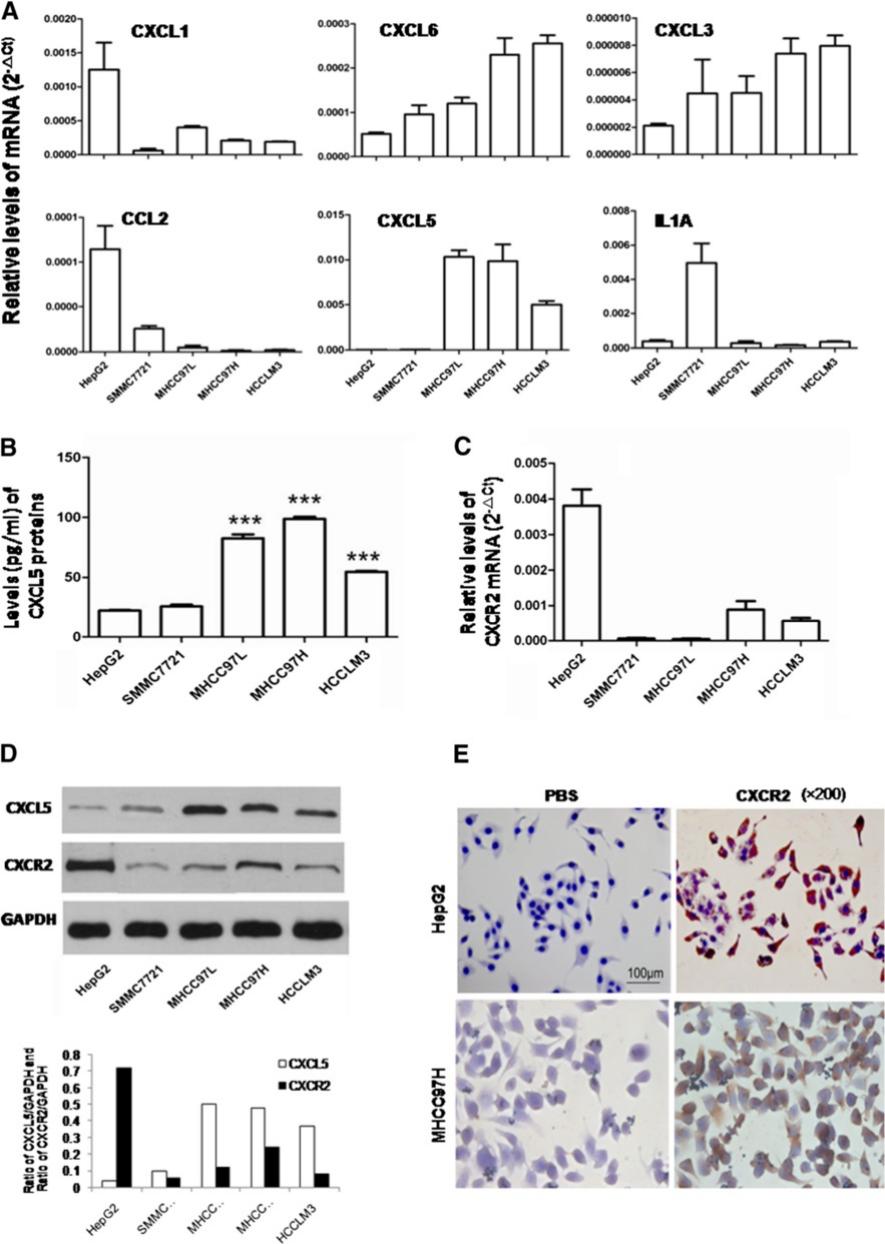
**Expression of inflammatory factors/receptors in LCCs. A**: Relative mRNA levels of CXCL1, CXCL3, CXCL5, CXCL6, CCL2 and IL-1A in five LCCs by RT-PCR; **B**: Levels of CXCL5 protein in cell culture supernatant by ELISA (***P < 0.001); **C**: Relative mRNA levels of CXCR2 in five LCCs by RT-PCR; **D**: Levels of CXCL5 and CXCR2 protein by Western Blot; **E**: Expression of CXCR2 protein by immunocytochemistry (×200).

We measured the expression levels of 89 inflammatory factors/receptors in HCCLM3 cells with high metastatic capacity and HepG2 cells with low metastatic capacity. The results showed that 18 factors were up-regulated in HCCLM3 including SPP1, CXCL5, C5, CXCL6, IL1RN, while 21 were down-regulated including C3, CXCL11, XCR1, CXCL10 (Table 1 and Additional file 2: Figure S1, not shown). Six chemokines (CXCL1, CXCL3, CXCL5, CXCL6, CCL2 and IL-1A) selected from the screening were validated in five LCCs with different metastatic capacities (in ascending order: HepG2, SMMC7721, MHCC97L, MHCC97H and HCCLM3). Apart from CXCL3, levels of CXCL1, CXCL5, CXCL6, CCL2 and IL-1A were consistent with the results of qRT-PCR array. Levels of CXCL5 in MHCC97L, MHCC97H and HCCLM3 cells were significantly higher than those in HepG2 and SMMC7721 (Figure 1A). We further found that levels of CXCL5 protein in MHCC97L, MHCC97H or HCCLM3 were higher than those from HepG2 or SMMC7721 through ELISA and Western Blot (P <0.001, Figure 1B and D). Expression of CXCR2 mRNA was high in HepG2 cells and medium in MHCC97H and HCCLM3, but not in SMMC7721 and MHCC97L (Figure 1C). Similar findings were noted in the expression of CXCR2 protein by Western Blot and immunocytochemistry (Figure 1D and E).

**Table 1:** qRT-PCR array (ratio) of inflammatory factors and receptors between HCCLM3 and HepG2

**Up-regulation**		**Down-regulation**	
**Gene**	**Fold change**	**Gene**	**Fold change**
CXCL12	2.3019	IL10RA	2.0307
IL13	2.3838	LTA	2.0895
IL36A	2.4158	ABCF1	2.197
IFNA2	3.2783	CXCL3	2.3116
CXCL8	3.4452	IL36G	2.6034
CARD18	4.2404	CXCL2	2.6868
IL1R1	5.9443	CXCL13	2.7931
TOLLIP	6.0692	RPL	2.809
CCL20	10.2342	CEBPB	3.1528
CCL15	10.7815	CCL5	3.1897
CCL26	13.5393	LTB4R	3.6407
CCL24	13.5841	B2M	3.8312
CXCL6	18.8126	IL-9	3.942
IL1RN	28.7457	TNF	4.9135
IL36B	34.1138	IL1A	5.7681
C5	114.9842	CXCL1	5.8887
CXCL5	1172.6051	CCL2	9.8094
SPP1	12470.4465	CXCL10	11.7757
		XCR1	35.7612
		CXCL11	58.2135
		C3	190.8385

Inflammatory factors & Receptors with fold change > 2 were shown.

### Chemotaxis of CXCL5 on LCCs

**Figure 2:**
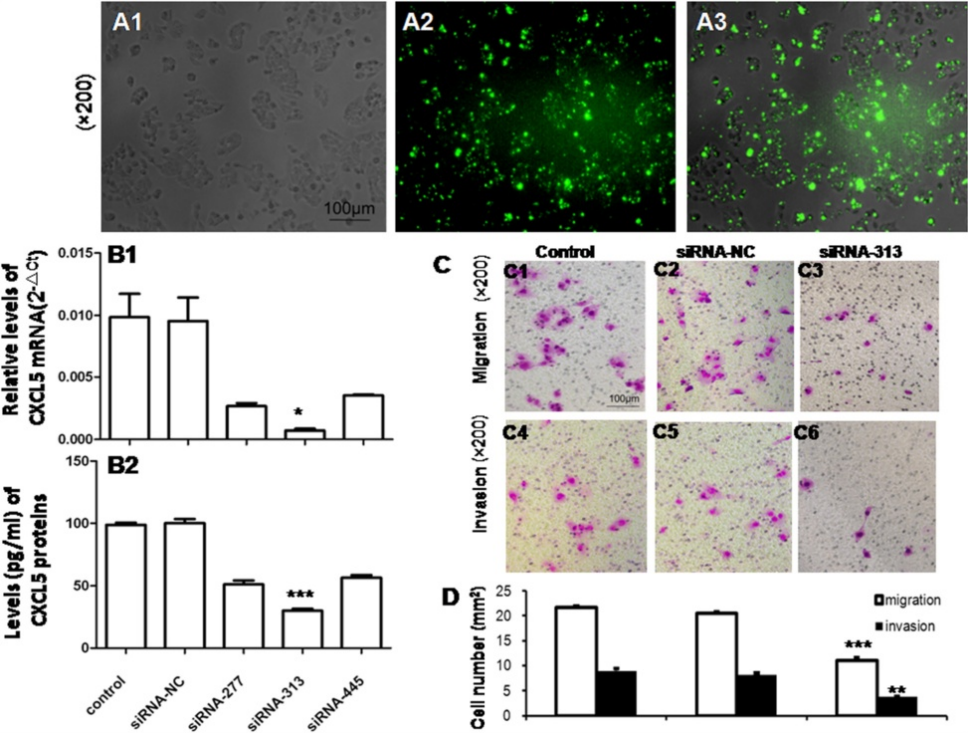
**Chemotaxis of CXCL5 on LCCs. A**: Transfection efficiency with the ratio of Lipofectame 2000 and siRNA at 1:20, including bright field **(A1)**, FAM **(A2)**, and merged **(A3)** (×200); **B**: Inhibitory efficiency of three siRNAs of CXCL5: relative mRNA levels of CXCL5 48hs after siRNA transfection measured by RT-PCR **(B1)** and protein levels of CXCL5 72hs after siRNA transfection measured by ELISA **(B2)**; **C**: Effect of CXCL5 on LCC migration and invasion measured by transwell assay (×200); **D**: Average cell numbers of migration and invasion in three identical experiments (*P < 0.05, **P < 0.01, ***P < 0.001).

**Figure 3:**
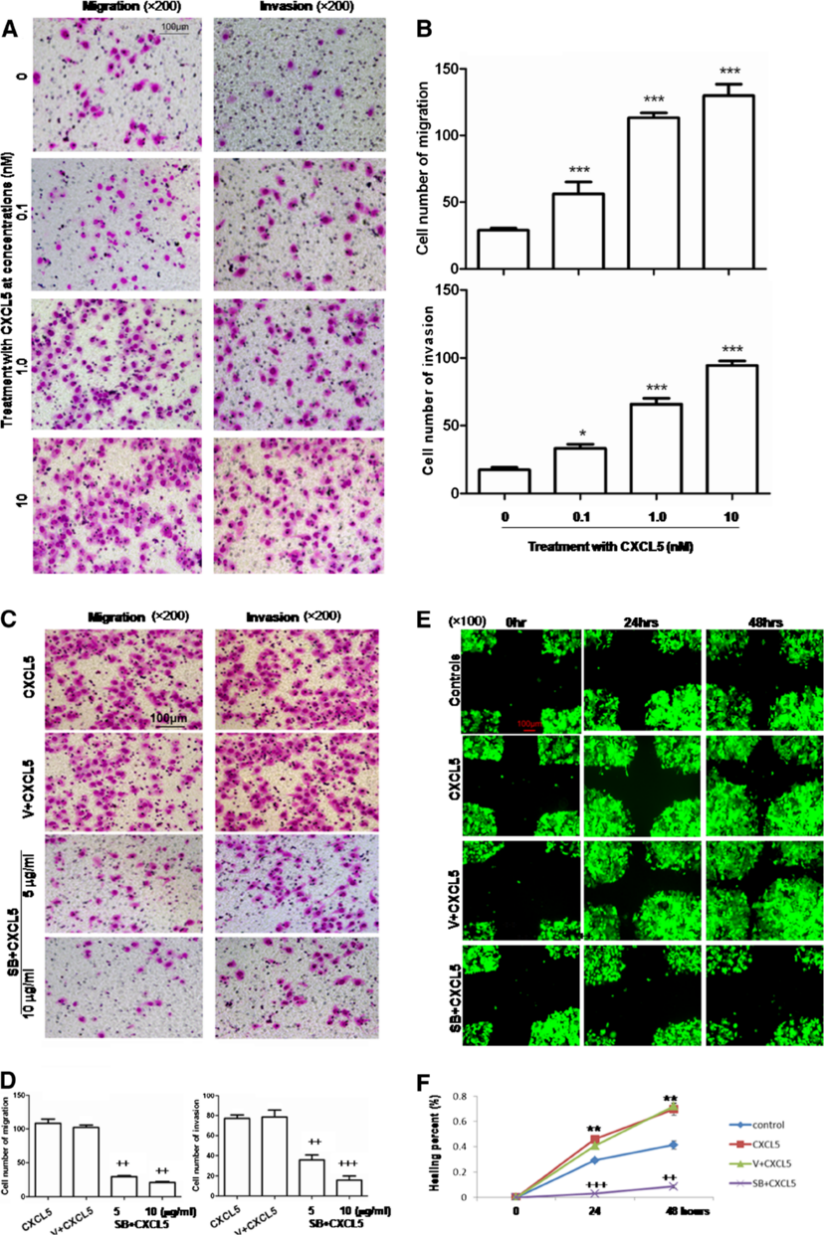
**Chemotaxis of recombinant CXCL5 on LCCs and Specificity of CXCL5/CXCR2. A**: Effect of recombinant CXCL5 on the migration and invasion of HepG2 measured by transwell assay (×200); **(B)** Average cell numbers of migration and invasion in three identical experiments (n = 3 each), HepG2 cells were incubated at the upper chamber and recombinant CXCL5 (0.1, 1.0, or 10 nM) was added into the lower compartment; **(C)** Effect of CXCR2 inhibitor SB225002 on CXCL5-induced migration and invasion of HepG2 measured by transwell (×200); **(D)** Average cell numbers of migration and invasion in three identical experiments (n = 3 each). **(E)** Effect of SB225002 on CXCL5-induced migration and invasion of HepG2 measured by wound-healing assay (×100); **(F)** Levels of healing percent. (*P < 0.05, **P < 0.01, ***P < 0.001, ++*P* < 0.01, +++*P* < 0.001).

We assessed the role of CXCL5 by transfecting siRNA into MHCC97H. The transfection efficiency was up to 70% with the ratio of Lipofectame2000 and siRNA at 1:20 (Figure 2A). Treatment with siRNA-277, siRNA-313 or siRNA-445 could inhibit 73, 93 or 64% of CXCL5 mRNA expression and 48, 70 or 43% of CXCL5 protein contents, respectively (Figure 2B). Cell migration and invasion were significantly inhibited 72 hrs after the transfection with siRNA-313 (P < 0.01, Figure 2C and D). We also assessed the effect of recombinant CXCL5 on the migration and invasion of HepG2 cells with high amount of CXCR2 and low metastatic capacity. The treatment with CXCL5 could increase the migration and invasion of HepG2 in a dose-dependent manner (*P* < 0.05 or less, Figure 3A and B).

### Specificity of CXCL5/CXCR2

Treatment with CXCR2 inhibitor SB225002 at 5 or 10 μg/ml could inhibit CXCL5-induced migration and invasion of HepG2 (Figure 3C). Migration and invasion of SB225002-treated cells were significantly lower than in CXCL5-stimulated cells (*P* < 0.01, respectively, Figure 3D). This was also evidenced by the measurement of healing percentage in Figure 3E. CXCL5 significantly increased the migration of HepG2 cells at both 24 and 48 hrs, as compared to controls, while the healing percentage of SB225002-treated cells was significantly lower than that of cells stimulated with CXCL5 (*P* < 0.01, respectively, Figure 3F).

### Role of CXCL5-induced signal pathway

**Figure 4:**
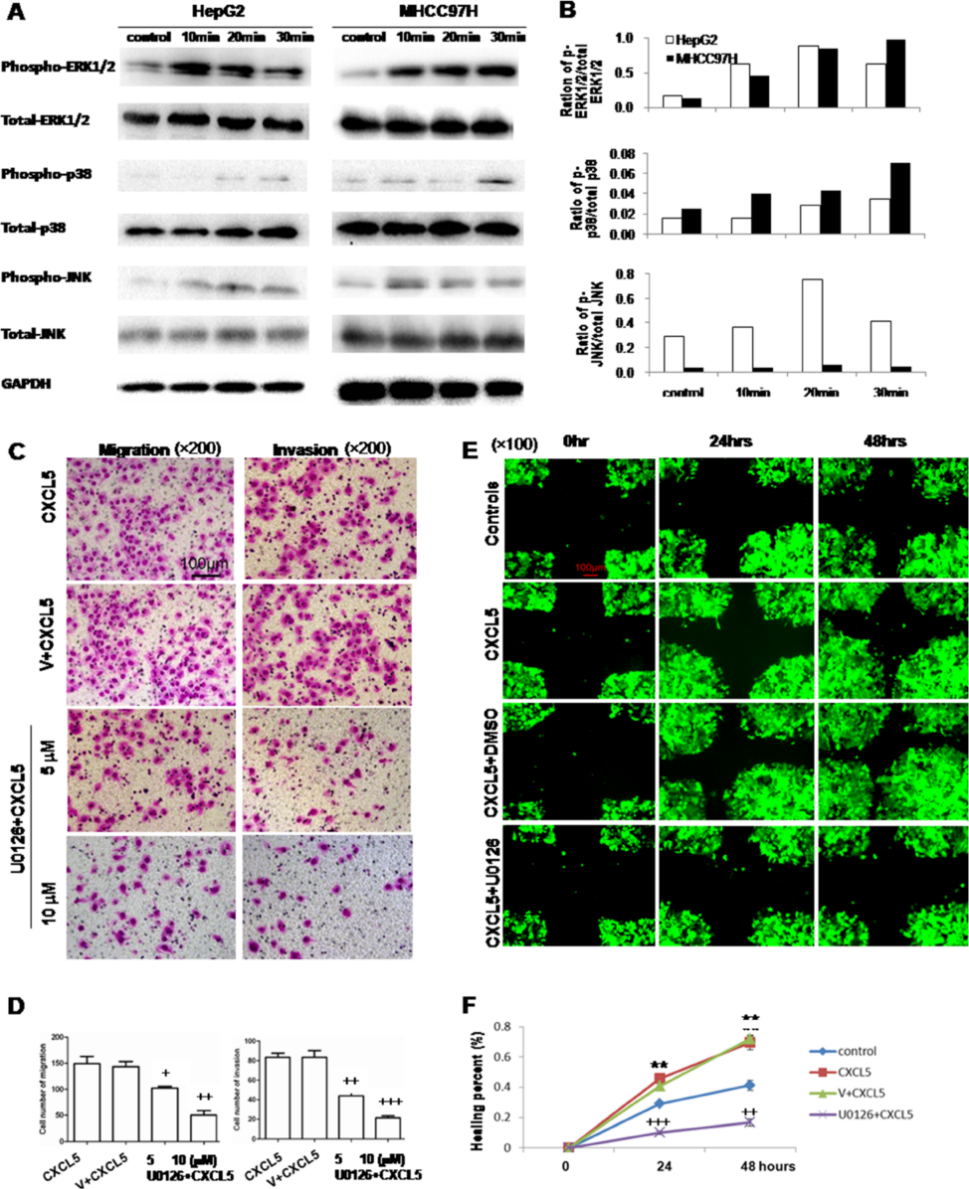
**Activation of ERK1/2, p38and JNK induced by CXCL5 and role of ERK1/2 signal pathway in the chemotaxis of CXCL5 on LCCs. (A)** Phosphorylated and total amounts of ERK1/2, p38 MAPK or JNK measured by Western blot in HepG2 and MHCC97H; **(B)** Ratio of p-ERK1/2/total ERK1/2, p-p38/total p38, p-JNK/total JNK in HepG2 and MHCC97H. **(C)** Effect of ERK1/2 inhibitor U0126 on CXCL5-induced migration and invasion of HepG2 measured by transwell assay (×200); **(D)** Average cell numbers of migration and invasion in three identical experiments (n = 3 each). **(E)** Effect of U0126 on CXCL5-induced migration and invasion of HepG2 measured by wound-healing assay (×100); **(F)** Levels of healing percent. GFP-HepG2 cells were pretreated with or without U0126 at 10 μM or DMSO for 2hs, followed by challenge with or without CXCL5 (controls) at 10 nM. (**P < 0.01, +*P* < 0.05, ++*P* < 0.01, +++*P* < 0.001).

The phosphorylated and total amounts of p38 MAPK, ERK1/2 or JNK were measured in HepG2 and MHCC97H cells cultured without serum for 24 hrs and then treated with CXCL5 at 10 nM for 10, 20 or 30 mins (Figure 4A). The amount of phosphorylated ERK1/2 increased steadily with a peak at 20 mins after the treatment with CXCL5 in HepG2, but increased continuously in MHCC97H (Figure 4B). The amount of phosphorylated p38 MAPK increased in both cells by time, while the peak of increased phosphorylated JNK was at 20 mins, as shown in Figure 4B.

HepG2 cells were pre-incubated with the ERK1/2 inhibitor U0126 in transwells for 2 hrs, followed by the stimulation of CXCL5 at 10 nM (Figure 4C) in order to further evaluate the role of ERK1/2 signal pathway in CXCL5-induced cell migration and invasion. U0126 at 5 or 10 μM significantly prevented CXCL5-induced cell migration and invasion in a dose-dependent pattern (*P* < 0.05 or less, Figure 4D). The inhibitory rates of U0126 at 5 and 10 μM were 32 and 66% in migration, and 46 and 74% in invasion, respectively. The inhibitory effects of U0126 on CXCL5-induced migration of HepG2 cells were further evidenced by the measurement of the wound healing percentage (Figure 4E). The healing percentage of cells stimulated with CXCL5 was significantly higher than those without stimulation, and significantly inhibited by U0126 at 24 and 48 hours (*P* < 0.01, Figure 4F).

## Discussion

The present study mapped out the mRNA expression of multiple inflammatory factors/receptors and demonstrated that SPP1 and CXCL5 were highly over-expressed in LCCs with high metastatic potentials. SSP1, also known as osteopontin, has been well documented by our institute as a promoter for hepatocellular carcinoma metastasis [16]. CXCL5 was also suggested to serve as a novel predictive marker for prognosis determination of many cancers, such as colorectal cancer [12]. It is further evidenced by recent studies that CXCL5 was over-expressed in HCC patients with shorter overall survival and high tumor recurrence [9]. Therefore, the present study aimed to provide more research about the important values and roles of CXCL5 in LCC migration and invasion.

The present study demonstrated that CXC chemokines with sequence Glu-Leu-Arg (ELR motif) were over-expressed in LCCs with high metastatic potentials, such as CXCL12, CXCL5, and CXCL8. The ELR^+^CXC chemokines have been proposed as important mediators of tumorigenesis, angiogenesis, progression, and indicators of prognoses in a number of cancers [17-21]. Out of those, higher expression and production of CXCL5 mRNA and protein were found in cells with high metastatic potentials. CXCL5 could also increase the migration and invasion of CXCR2-positive LCCs. Thus LCCs might act as the producer and receptor of CXCL5 in the development and metastasis of the cancer and may themselves play a critical role in the initiation and formation of inflammatory microenvironment like other cancer cells [22]. In addition, our data also suggested both autocrine and paracrine mechanisms and manners of CXCL5 in LCCs. CXCL5 could be produced by liver cancer cells, but also by inflammatory cells (e.g. neutrophil, monocyte, macrophage) and structure cells (e.g. epithelial, endothelial, fibroblastic) [8]. Autocrine and paracrine CXCL5 may directly chemo-attract liver cancer cells and circulating leukocytes for the development of an inflammatory microenvironment.

CXCR2 [interleukin (IL)-8R] is a member of the G-protein–coupled receptor superfamily, and the receptor of ELR^+^CXC chemokines. CXCR2 expression in endothelial cells is activated by ELR^+^CXC chemokines and promotes tumor growth [23]. CXCR2 was also documented to be correlated with intrahepatic metastasis, portal cancer embolus and TNM staging of liver cancer patients [20,24-27], although the role of CXCR2 in tumor cells is debated. Activated CXCR2 was found to promote cell proliferation, migration, and invasion [28] and to assist cancer cells in evading stress-induced apoptosis [29]. On the other hand, the depletion of CXCR2 delays the replicative senescence and impairs the senescence response to oncogenic signals [30], suggesting that it acts as a tumor suppressor. However, our results have shown that the expression of CXCR2 was higher in HepG2 cells than that in MHCC97H and HCCLM3, which indicated CXCR2 might be related to the metastatic capacity of LCCs. However, the results do not imply CXCR2 is a tumor suppressor, and further studies on the role of CXCR2 per se will be summarized in our next study. Notwithstanding its limitations, our results did indicate that the certain expression of CXCR2 might be the foundational requirement for LCC migration and invasion when the concentration of CXCL5 in the environment was high enough. CXCR2 expression in HepG2 with lower metastatic potential is activated by CXCL5, and promotes the migratory and invasive capacity of HepG2. Moreover, the chemotaxis induced by CXCL5 is CXCR2-dependent, evidenced by the inhibitory effects of SB225002. Thus, CXCL5-CXCR2 may play an important role in LCC metastasis.

Cancer invasion and metastasis is initiated and maintained by signaling pathways in the tumor microenvironment, followed by cell migration into the adjacent tissue [31,32]. Invasion- and migration-promoting chemokines and corresponding receptors mediate invasive migration of tumor cells through a variety of pathways [33]. Our data demonstrated that the activations of ERK1/2, p38 MAPK and JNK pathways were involved in the regulation of CXCL5-induced migration and invasion of LCCs. Of those, the ERK1/2 signal pathway might play a more dominate role in the movement of LCCs with CXCL5 stimulation, evidenced by the finding that U0126 at 10 μM could inhibit about 65 and 75% of CXCL5-induced cell migration and invasion.

**Figure 5:**
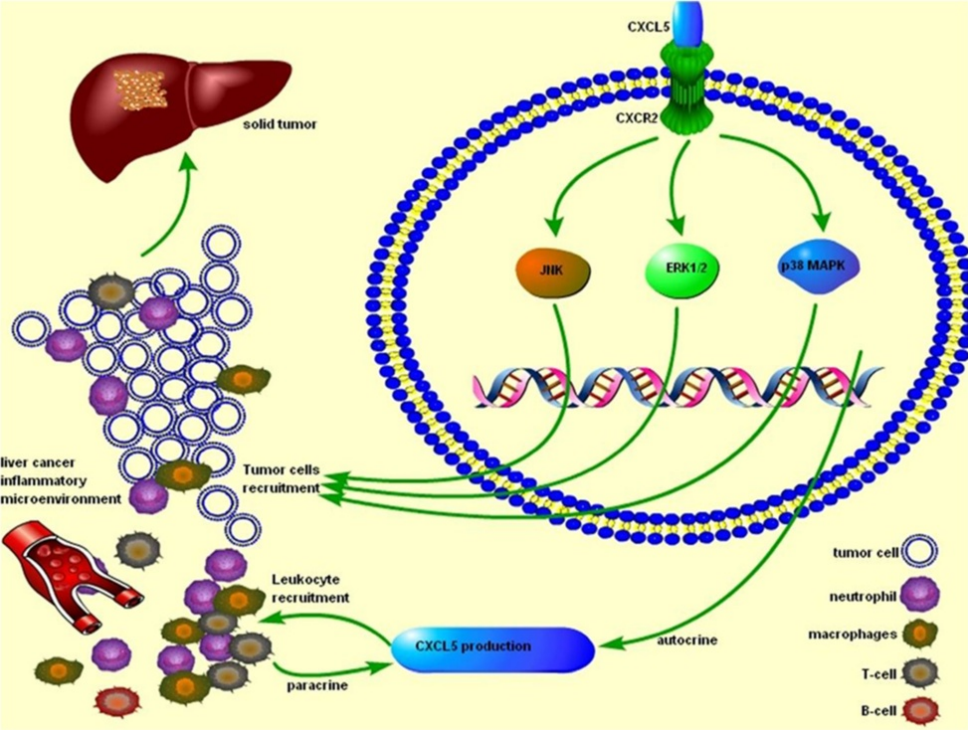
**Roles of CXCL5 in the metastasis of liver cancer cells.** CXCL5 increased LCCs migration and invasion, probably through autocrine and paracrine mechanism. CXCL5-CXCR2-ERK1/2 pathways could play critical and necessary roles in the regulation of LCCs migration. LCCs per se act as the producer and receptor of CXCL5, responsible for the development of inflammatory microenvironment and the promotion of malignancy.

In conclusion, the present study confirmed the over-expression of CXCL5 in LCCs with high metastatic potentials and that CXCL5 could increase LCC’s migration and invasion, probably through autocrine and paracrine mechanisms. Evidence also suggests that CXCL5-CXCR2-ERK1/2 pathways might play critical roles in LCC migration and invasion (Figure 5) and Additional file 3: Figure S2, not shown).

## Competing interests

The authors declare that they have no competing interests.

## Authors’ contributions

XX and PH made the study plan and performed the experimental studies, data analysis, and manuscript writing. BY performed the measurement of cell migration and invasion. XW and JX made the study plan, data analyses, drew the figures and wrote the manuscript. All authors read and approved the final manuscript.

## Supplementary Material

Additional file 1: Table S1 Sequences mentioned in the article. Click here for file

Additional file 2: Figure S1 qRT-PCR array (ratio) of inflammatory factors and receptors between HCCLM3 and HepG2. Click here for file

Additional file 3: Figure S2 The work flow of this research. Click here for file
