# Supplementary material for: Roles of CXCL5 on migration and invasion of liver cancer cells
Source: J Transl Med. 2014 Jul 10;12:193. doi: 10.1186/1479-5876-12-193 (PMC4097051; doi:10.1186/1479-5876-12-193)
Supplement: Additional file 1: Table S1 — Sequences mentioned in the article. [file 1479-5876-12-193-S1.doc]

**Additional file 1: Table S1 Sequences mentioned in the article**

| **Name** | **Sequences** | |
| --- | --- | --- |
|  | **Forward primer** | **Reverse primer** |
| **CXCL1** | 5’-ATGGCCCGCGCTGCTCTC-3’ | 5’-CGGGGGACTTCACGTTCACACTT-3’ |
| **CXCL3** | 5’-TGCTGCTCCTGCTCCTGGTG-3’ | 5’-AGTTGGTGCTCCCCTTGTTCA-3’ |
| **CXCL5** | 5’-GAGAGCTGCGTTGCGTTTGTTTAC-3’ | 5’-CCGTTCTTCAGGGAGGCTACCA-3’ |
| **CXCL6** | 5’-GTCCTTCGGGCTCCTTGTGC-3’ | 5’-GGGGCTTCCGGGTCCAGA-3’ |
| **CCL2** | 5’-AGTCTCTGCCGCCCTTCTGTG-3’ | 5’-CTTCGGAGTTTGGGTTTGCTTGTC-3’ |
| **IL-1A** | 5’-TCGCCAATGACTCAGAGGAAGAAA-3’ | 5’-ATGTAATGCAGCAGCCGTGAGGTA-3’ |
| **β-actin** | 5’-CGTGGGCCGCCCTAGGCACCA-3’ | 5’-TTGGCTTAGGGTTCAGGGGGG-3’ |
| **siRNA-227** | 5‘--GACCACGCAAGGAGUUCAUTT--3’ | 5‘--AUGAACUCCUUGCGUGGUCTT--3’ |
| **siRNA-313** | 5‘--UCUGCAAGUGUUCGCCAUATT--3’ | 5‘--UAUGGCGAACACUUGCAGAT--3’ |
| **siRNA-445** | 5‘--UGGAAACAAGGAAAACUGATT--3’ | 5‘—UCAGUUUUCCUUGUUUCCATT--3’ |
| **siRNA-NC** | 5’--UUCUCCGAACGUGUCACGUTT --3’ | 5‘--ACGUGACACGUUCGGAGAATT --3’ |

siRNA-227, -313, -445 are three kinds of CXCL5-siRNAs targeted on different sequences. siRNA-NC is a negative control siRNA with the same nucleotide composition but which lacks significant sequence homology to CXCL5.
