# Supplementary figures and images for: Roles of CXCL5 on migration and invasion of liver cancer cells
Source: J Transl Med. 2014 Jul 10;12:193. doi: 10.1186/1479-5876-12-193 (PMC4097051; doi:10.1186/1479-5876-12-193)

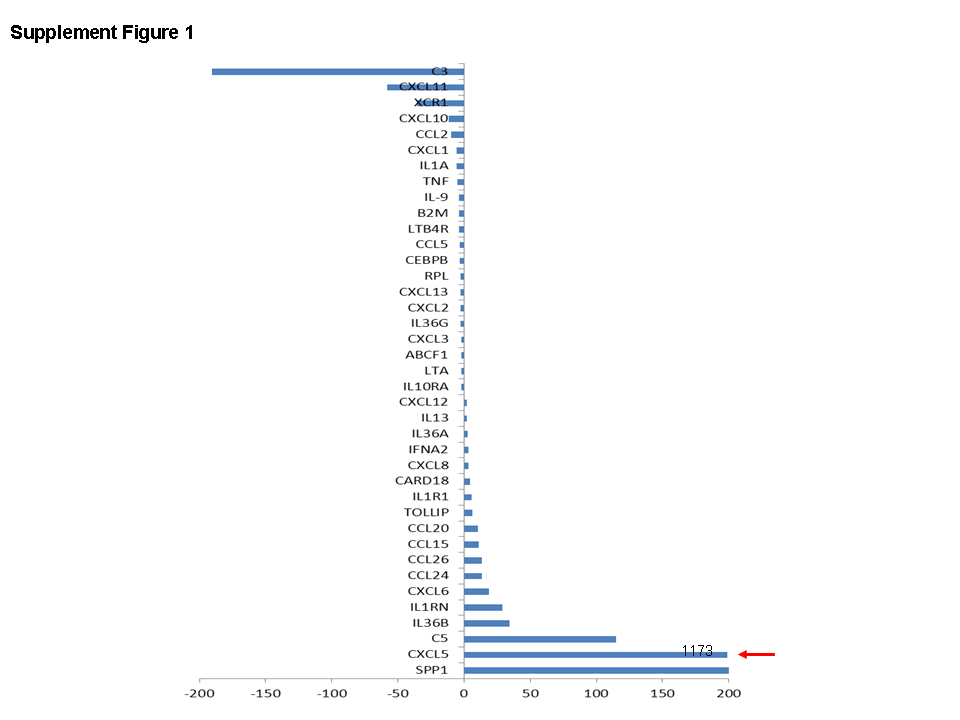

Supplement: Additional file 2: Figure S1 — qRT-PCR array (ratio) of inflammatory factors and receptors between HCCLM3 and HepG2. [file 1479-5876-12-193-S2.tiff]

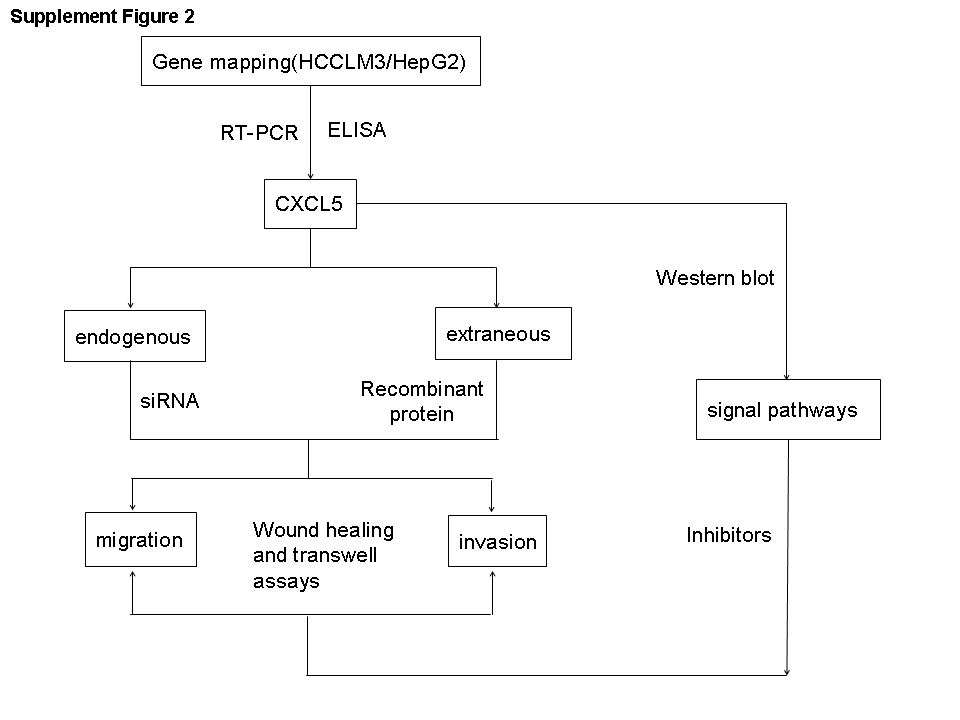

Supplement: Additional file 3: Figure S2 — The work flow of this research. [file 1479-5876-12-193-S3.tiff]
